# Activation of G protein-coupled estrogen receptor 1 induces coronary artery relaxation via Epac/Rap1-mediated inhibition of RhoA/Rho kinase pathway in parallel with PKA

**DOI:** 10.1371/journal.pone.0173085

**Published:** 2017-03-09

**Authors:** Xuan Yu, Qiao Zhang, Yan Zhao, Benjamin J. Schwarz, John N. Stallone, Cristine L. Heaps, Guichun Han

**Affiliations:** 1 Department of Physiology and Pharmacology, Texas A&M University, College Station, TX, United States of America; 2 Department of Cardiovascular Surgery, Union Hospital, Tongji Medical College, Huazhong University of Science and Technology, Wuhan, China; 3 Department of Cardiovascular Medicine, First Affiliated Hospital of Xi’an Jiaotong University, Xi’an, China; 4 Women's Health Division, Michael E. DeBakey Institute, Texas A&M University, College Station, TX, United States of America; BloodCenter of Wisconsin, UNITED STATES

## Abstract

Previously, we reported that cAMP/PKA signaling is involved in GPER-mediated coronary relaxation by activating MLCP via inhibition of RhoA pathway. In the current study, we tested the hypothesis that activation of GPER induces coronary artery relaxation via inhibition of RhoA/Rho kinase pathway by cAMP downstream targets, exchange proteins directly activated by cAMP (Epac) as well as PKA. Our results show that Epac inhibitors, brefeldin A (BFA, 50 μM), or ESI-09 (20 μM), or CE3F4 (100 μM), all partially inhibited porcine coronary artery relaxation response to the selective GPER agonist, G-1 (0.3–3 μM); while concurrent administration of BFA and PKI (5 μM), a PKA inhibitor, almost completely blocked the relaxation effect of G-1. The Epac specific agonist, 8-CPT-2Me-cAMP (007, 1–100 μM), induced a concentration-dependent relaxation response. Furthermore, the activity of Ras-related protein 1 (Rap1) was up regulated by G-1 (1 μM) treatment of porcine coronary artery smooth muscle cells (CASMCs). Phosphorylation of vasodilator-stimulated phosphoprotein (p-VASP) was elevated by G-1 (1 μM) treatment, but not by 007 (50 μM); and the effect of G-1 on p-VASP was blocked by PKI, but not by ESI-09, an Epac antagonist. RhoA activity was similarly down regulated by G-1 and 007, whereas ESI-09 restored most of the reduced RhoA activity by G-1 treatment. Furthermore, G-1 decreased PGF2α-induced p-MYPT1, which was partially reversed with either ESI-09 or PKI; whereas, concurrent administration of ESI-09 and PKI totally prevented the inhibitory effect of G-1. The inhibitory effects of G-1 on p- MLC levels in CASMCs were mostly restored by either ESI-09 or PKI. These results demonstrate that activation of GPER induces coronary artery relaxation via concurrent inhibition of RhoA/Rho kinase by Epac/Rap1 and PKA. GPER could be a potential drug target for preventing and treating cardiovascular diseases.

## Introduction

G-protein-coupled estrogen receptor 1 or GPER is emerging as therapeutic target for the treatment of CVD [1]. GPER activation reduces blood pressure, heart and brain infarction [2, 3], and relaxes peripheral blood vessels [4, 5]. Moreover, selective activation of GPER relaxes porcine coronary arteries [6–8]. The mechanism of GPER-mediated vascular relaxation is, however, far from clear. As a typical G-protein-coupled receptor, GPER has been reported to interact with Gs, and thereby activate adenylyl cyclase and increase cAMP production in GPER-transfected HEK293 cell plasma membrane extracts and human CASMCs [9, 10]. Our recent work has demonstrated that cAMP/PKA signaling is involved in GPER-mediated relaxation. In human and porcine CASMCs, GPER activation increased cAMP production and activated PKA activity, which in turn, phosphorylated RhoA and thus, inhibited RhoA activity, resulting in activation of the myosin light chain (MLC) phosphatase (MLCP) and dephosphorylation of MLC [10].

The newly-discovered target of cAMP, exchange proteins directly activated by cAMP (Epac), has been revealed to be a novel downstream mechanism for cAMP to govern signaling in the cardiovascular system and other tissues [11, 12].Its primary function is to act as guanine nucleotide exchange factors (GEF) for Rap GTPases—which act as molecular switches that cycle between an active GTP-bound state and an inactive GDP-bound state [13]. It has been reported that an Epac agonist induces pulmonary and portal vein relaxation by activation of MLCP via Rap1 inhibition of Rho kinase activities [14]. In this study, we explored the role of Epac and its downstream signaling in mediating GPER-induced coronary artery relaxation.

## Materials and methods

### Tension studies

Fresh porcine hearts were obtained from a local abattoir K&C Meat Processing, the geographic coordinates are latitude 30.372080° and longitude -96.070557°. The hearts were immediately placed in cold Dulbecco’s Phosphate Buffered Saline (Sigma) and transported back to the laboratory. Left anterior descending (LAD) coronary arteries were dissected free of fat and connective tissue, and cut into rings (axial length ~ 5 mm) to be used in isometric contractile force recordings. To eliminate effects of endothelium-derived vasoactive factors, artery rings were endothelium-denuded by removing the endothelium (i.e., gently rubbing the intimal surface with cotton strings). Only the rings with successful endothelium denudation were used, which was confirmed by the absence of relaxation to bradykinin (100 nM) exposure. Arterial rings were mounted on the two wires of isometric myographs (Danish Myograph Technology) filled with 10 ml modified Krebs-Henseleit buffer (in mM): 122 NaCl, 4.7 KCl, 15.5 NaHCO_3_, 1.2 KH_2_PO_4_, 1.2 MgCl_2_, 1.8 CaCl_2_, 11.5 glucose, pH 7.2, bubbled with 95% O_2_−5% CO_2_ (pH 7.4) at 37°C. One wire was connected to a force-displacement transducer and the other to a stationary micrometer. The equilibration time for the preparations in Krebs-Henseleit buffer was 90 min. The optimal resting tension was set at 20 mN in the first 30 min by gradually stretching the artery rings as in our previous work [6, 10]. Isometric tension was recorded by using the LabChart data acquisition system (AD Instruments) on a PC computer. The preparations were contracted, washed and allowed to relax to basal tension for 3 times with PGF2α (1 μM). Then PGF2α (1 μM) was used to induce a stable contraction and G-1 was added in a cumulative manner by increasing the concentration in log increments. Pharmacological inhibitors were applied 30 min prior to measurement of vasodilator concentration-response curve. In each set of experiments, one ring was exposed only to the constrictor agent PGF2α (1 μM) and vehicle as time control for potential fading of the contractile response. The vehicle (dimethylsulfoxide, DMSO) was added in cumulative manner by adding the identical amount of DMSO used for each concentration of G-1, 8-(4-chlorophenylthio)-2-O-methyladenosine-3,5-cyclicmonophosphate, 8-CPT-2Me-cAMP (007). The total amount of vehicle was less than 0.1% of the tissue bath volume. Relaxation responses were calculated as the % reduction in tension at each drug concentration from the precontracted state.

### Vascular smooth muscle cell culture

The primary porcine CASMCs culture was established as described previously [15, 16]. Briefly, coronary arteries were isolated from porcine hearts. After removing the endothelial layer with a cotton-tipped swab, the media layer was dissected free from the adventitia. The medial layer was then cut into small pieces and moved into a 25-cm^2^ tissue culture flask containing 10 ml dissociation medium (in mM):110 NaCl, 5 KCl, 2 MgCl_2_, 0.16 CaCl_2_, 10 HEPES, 10 NaHCO_3_, 0.5 KH_2_PO_4_, 0.5 NaH_2_PO_4_, 0.48 EDTA, 10 taurine, and 10 glucose, with addition of 24 mg elastase, 6 mg collagenase, and 15% bovine serum. After gently shaking at 37°C for 3 hours, cells were then dispersed by gentle trituration and centrifuged at 800 rpm for 5 min. The pellet was resuspended with CASMC medium, SmGM (Lonza Corp.) and cells were seeded in a 25-cm^2^ tissue culture flask coated with 1% gelatin and incubated at 37°C in a humidified 5% CO_2_ incubator. The purity of porcine CASMCs was verified by positive staining with smooth muscle-specific α-actin [17]. Passage 4 and 5 of porcine CASMCs were used in this study.

### Western blot

Phosphorylation of MYPT1 (pMYPT1), the regulatory subunit of MLCP, from porcine coronary artery tissue lysates, phosphorylation of MLC (p-MLC), vasodilator-stimulated phosphoprotein (pVASP), and p-RhoA from porcine CASMC lysates were detected by Western blot analysis as previously described [10]. Briefly, arterial rings were prepared as in isometric tension studies and mounted in the chamber of the myograph, equilibrated, and contracted three times with PGF2α (1 μM) as described above. Artery rings were pretreated with agonist in the absence or presence of antagonists for 30 min before exposure to PGF2α (1 μM). The arterial rings were collected at the contraction plateau and snap frozen in liquid nitrogen. Tissues were pulverized and lysed in homogenization buffer, the composition of the buffer is (mM): 50 Tris–HCl, 0.1 EGTA and 0.1 EDTA, with 0.1% SDS, 1% NP-40, and 0.1% deoxycholic acid. For detection of phosphorylation of MLC20 and VASP, porcine CASMC cells were treated with agonists and antagonists as indicated in the results. Cells were then harvested and homogenized. Protein concentrations were determined by using detergent-compatible colorimetric assay kit (Bio-Rad). Proteins were separated by using a Mini Protean II SDS-PAGE gel kit (Bio-Rad) according to the manufacturer’s instructions.

After separation, proteins were transferred to Hybond enhanced chemiluminescence (ECL) membrane (Amersham Pharmacia Biotech) with a Mini-Trans-Blot Electrophoretic Transfer Cell (Bio-Rad) at constant voltage of 100 V for 1 hour. Then membranes were blocked with 5% fat free milk for 1 hour at room temperature and rinsed with Tris-buffered saline (TBS)-Tween 20 (TBST) three times, 5 min per wash. Membranes were then probed with specific primary antibodies in TBST containing 5% fat free milk overnight at 4°C (the dilutions of antibodies: p-MYPT1, Thr-853 p-MYPT1, 1:1000, and p-RhoA, ser 188, 1:200 from Santa Cruz Biotechnology; p-MLC, Ser19, 1:1000, and p-VASP, Ser157, 1:1000 from Cell Signaling). The β-actin primary antibody (dilution: 1:2000 from Santa Cruz Biotechnology) was used for protein loading control in the protein band densitometry analysis. Experiments were performed at least in triplicate, and mean values ± SEM were calculated and graphed.

### Rap 1 activation assay

The Rap1 activation assay is based on the differential affinity of Rap1-GTP and Rap1-GDP for the Rap binding domain of Ral GDS as described in the manufacturer’s protocol. Rap1 activity was determined by using a Rap1 activation kit (Upstate Biotechnology). Briefly, porcine CASMCs were treated with G-1 (1 μM) between 2 and 15 min with or without ARF-GEF inhibitor, brefeldin A (BFA, 50 μM), a potential Epac inhibitor [18] and 007 (50 μM) for 5 min. Cell lysates were equally split into two microfuge tubes, then incubated with GTPγS (1 mM) in one tube and GDP (1mM) in the other tube. Then Ral GDS-RBD agarose was added to each tube. Following 45 min incubation at 4°C, beads were rinsed three times with ice-cold lysis buffer and proteins were eluted from the beads in Laemmli reducing sample buffer and then detected with Western blot as described above by using specific anti-Rap1 polyclonal antibody which recognizes recombinant human Rap1a and Rap1b proteins.

### Rap1 small interfering RNA (siRNA) transfection

Small interfering RNA (siRNA) specific for rap1 and scrambled siRNA used as a control were obtained from Santa Cruz biotechnology (Santa Cruz, CA). Porcine CASMCs, passage 4–5, were seeded in 60mm culture dishes at a density of 3 × 10^5^ cells/dish. After overnight incubation, cells were transfected with the rap1 siRNAs (100 pmol/dish) or scrambled RNA by using Lipofectamine RNAiMAX Reagent (Life Technologies, Inc.) and then incubated for 6 hours, according to the manufacturer’s instructions. The transfection medium was replaced with SmGM complete medium (Lonza Corp.) and the cells were incubated for another 48 hours. Then after serum-deprived for 24 hours, the cells were treated with 0.1% DMSO (as solvent control) or G-1 (1 μM). Then cells were harvested, centrifuged at 4°C and 10000 rpm for 10 min and supernatant was removed to clean tubes for Western blot detection of RhoA Phosphorylation and knockdown of total rap1. Beta-actin served as loading control.

### RhoA activation assay

Porcine CASMCs, passage 4–5, were serum- deprived 24 hours after cells reached approximately 50% confluence. After incubated with 0.1% DMSO (as solvent control), PGF2α (1 μM), PGF2α (1 μM) +G-1 (1 μM), and PGF2α (1 μM) +G36 (an antagonist of GPER, 5 μM) + G-1(1 μM), cells were harvested, centrifuged at 4°C and 10000 rpm for 10 min and the supernatant was transferred to a clean tube. Active Rho A- GTP activity was measured using RhoA G-LISA® kit (Cytoskeleton), a direct measurement of RhoA activity [19], according to the manufacturer’s instructions. Briefly, cell lysates were equalized according to the total protein and loaded into a Rho-GTP binding 96 -well plate, incubated with Anti-RhoA antibody and then secondary antibody. Absorbance was read at 490 nm. Active RhoA was normalized to total RhoA to compare RhoA activity between groups.

### Statistical analysis

In tension studies, relaxation responses to agonists were compared in the absence and presence of selective antagonist or the solvent control. Different treatments with cumulative concentrations were analyzed by using two-way analysis of variance (ANOVA). Data were presented as mean percent relaxation, with the standard error of the mean (SEM). The number of experiments is indicated in parentheses. Statistical differences were analyzed with Prism program (GraphPad Software Inc., San Diego, CA). For immunoblot analysis, one-way ANOVA was used to detect significant differences among all treatments and student’s t-test was used in paired groups. Bonferroni correction was used to correct for type 1 error associated with multiple comparisons. P value ≤0.05 was considered as significant.

### Drugs

G-1 was purchased from Calbiochem. 8-(4-chlorophenylthio)-2-O-methyladenosine-3,5-cyclicmonophosphate [8-CPT-2Me-cAMP (007)] and 3- [5- (tert-Butyl) isoxazol- 3- yl]- 2- [2- (3- chlorophenyl)hydrazono]- 3- oxopropanenitrile (ESI-09) was from Biolog Life Science Institute. PKI (14–22) amide (myristoylated) was obtained from Enzo Life Sciences. Brefeldin A was from LC laboratories and farnesyl thiosalicylic acid was from Cayman Chemical. All other chemicals were purchased from Sigma-Aldrich Corporation.

## Results

### Epac and coronary artery relaxation

**Fig 1:**
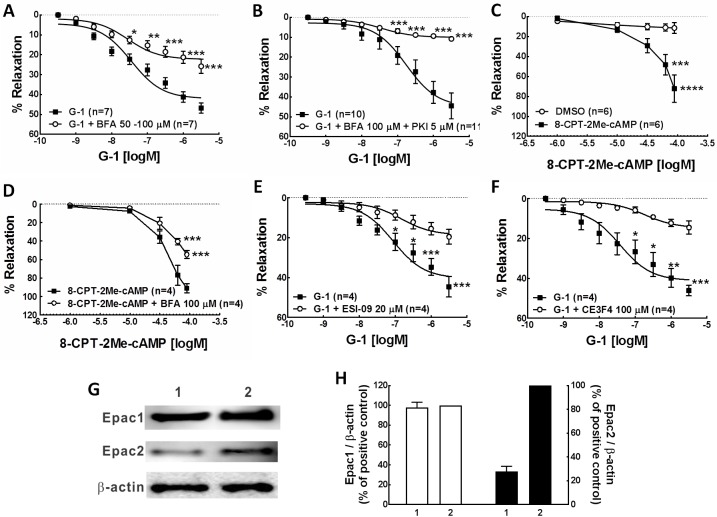
Epac contributes to G-1-induced relaxation of coronary arteries. Concentration-response relationship for G-1- or 8-CPT-2Me-cAMP -induced relaxation in endothelium denuded, PGF2α (1 μM) precontracted coronary artery were observed in the presence or absence of Epac inhibitors. A & B: in the presence or absence of Epac inhibitor, BFA (A); in the presence or absence of both Epac and PKA inhibitors, BFA and PKI (B). C&D: Concentration-response relationship for Epac agonist 8-CPT-2Me-cAMP (007)-induced porcine coronary artery relaxation in the absence (C) or presence (D) of BFA pretreatment. E & F: Concentration-response relationship for G-1-induced relaxation in the presence or absence of the Epac inhibitors, ESI-09 (E) or CE3F4 (F). Each point represents the mean percent relaxation effect ± SEM., *** p<0.001, compared to G-1 group or solvent control. G. A representative Western blot of Epac1 and Epac2 expression. Well1 is the loading of porcine coronary artery tissue lysates. Well2 is the loading of rat brain tissue lysates, serving as a positive control. Beta-actin was used as a protein loading control. H. A summary bar graph of densitometry analysis of Epac1 and Epac2 Western blot bands.

The pretreatment of artery rings with the ARF-GEF inhibitor brefeldin A (BFA, 50 μM), a proven Epac inhibitor, significantly attenuated G-1-induced porcine coronary artery relaxation, the reduced relaxation was 44.90% of the total relaxation effect of G-1 (Fig 1A, Table 1), similar to the effect of PKI (14–22) amide (5 μM), a PKA inhibitor, in our previously study [10]. When artery rings were pretreated with both BFA (50 μM) and PKI (5 μM), the inhibition of G-1-induced relaxation response reached to 76.98% (Fig 1B, Table 1), suggesting an additive effect of Epac and PKA. As expected, the 8-CPT-2Me-cAMP (007), an analog highly selective for activation of Epac [20], caused a concentration-dependent relaxation of coronary artery rings (Fig 1C, Table 1). However, the significant relaxation occurred at much higher concentration (EC50 = 49.4 μM), compared to G-1 (EC50 = 0.027 μM), but it was similar to the effect of PKA agonist 6-Bnz-cAMP in our previous study [10]. As expected, the concentration-dependent relaxation response of 007 was markedly inhibited by brefeldin A (50 μM), with no change of EC50 (49.4 μM without brefeldin A and 49.5 μM with brefeldin A) (Fig 1D and Table 1). We then further tested ESI09, a specific Epac1 and Epac2 inhibitor [21] and CE3F4, an uncompetitive inhibitor of Epac1 [22] in the construction of concentration-response relationship curve of G-1 relaxation experiments. As expected, both ESI-09 (20 μM) and CE3F4 (100 μM) have significantly attenuated the relaxation effect of G-1, CE3F4 has the most inhibition effect among these three inhibitors of Epac (Fig 1E & 1F, Table 1). It could be because Epac1 is the predominant expression form of Epac in porcine coronary arteries shown by Western blot result (Fig 1G). Together, these results demonstrate that Epac is involved in the G-1 induced coronary artery relaxation.

**Table 1:** Effects of compounds on porcine coronary artery relaxation response to G-1.

Compounds	% reduction of the G-1 relaxation	% relaxation	EC_50_ (μM)
G-1 ^+^		46.78 ± 2.52 (n = 10)	0.027
G-1 + BFA 50–100 μM	44.90%	25.78 ± 3.51 (n = 7) ***	0.028
G-1 + BFA 100 μM + PKI 5 μM	76.98%	10.77 ± 0.87 (n = 11) ***	0.174
G-1 + ESI-09 20 μM	58.12%	19.59 ± 3.73 (n = 4) ***	0.146
G-1 + CE3F4 100 μM	68.98%	14.51 ± 3.35 (n = 4) ***	0.028
DMSO ^++^		11.39 ± 5.31 (n = 6)	5.56
8CPT-2Me-cAMP (007) 90 μM ^+++^		72.10 ± 13.55 (n = 6) ****	49.4
8CPT-2Me-cAMP (007) 90 μM + BFA 100 μM ^++++^		54.59 ± 4.22 (n = 4) ***	49.5

Values are given as mean ± SEM. The number of experiments is indicated in parentheses.

***P<0.001

****P<0.0001, significant difference compared with control by using two-way ANOVA.

In rows 1–5 artery rings were pretreated with each of the inhibitors and the results were compared to G-1 (3 μM) alone group (+G-1).

In rows 6–8

^++^DMSO group was used as solvent control.

^+++^8-CPT-2Me-cAMP (007) was used as vasorelaxant agents and the result was compared to DMSO group.

^++++^Artery rings were pretreated with BFA, then relaxed with 8-CPT-2Me-cAMP (007) and the result was compared to 8-CPT-2Me-cAMP (007) alone group.

### Rap1 and VASP

**Fig 2:**
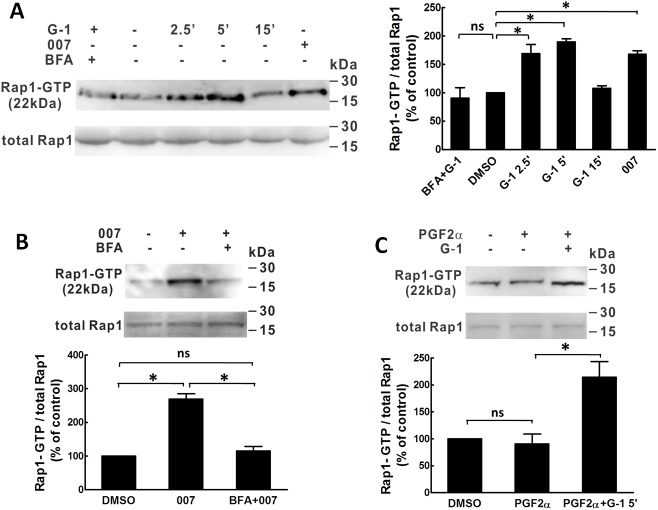
G-1 stimulates Rap1 activities in porcine coronary artery. Rap1 activity measured with a Rap1 activation kit. Porcine coronary artery SMCs (passage 4 and 5) were serum deprived for 24 hours, then treated with: BFA (50 μM) + G-1 (1 μM); G-1 (1 μM) for 0, 2.5, 5, and 15 min; 007 (100 μM) (A); PGF2α (1 μM) for 10 min, PGF2α (1 μM) for 10 min + G-1(1 μM) for 5 min (B); 007 (50 μM) for 5 min, BFA (50 μM) for 10 min + 007 (50 μM) for 5min (C); DMSO (0.1%) was used as solvent control. SMC lysate in Rap1 activation lysis buffer, upper band: cell lysate was pre-incubated with GTPγS prior to precipitation with Ral GDS-RBD; Lower band: cell lysate was pre-incubated with GDP prior to precipitation with Ral GDS-RBD. Left panel in 2A and upper panels in 2B-2C are a representative Western blot bands of Rap1-GTP and total Rap1 detected by using anti-Rap1 antibody from 3 individual assays. Rap1-GTP is the active form of Rap1. The lower panel is a summary bar graph of the Rap1 Western blot bands analyzed by densitometry. Rap1-GTP/total Rap1 ratio was normalized to control % (0 min of G-1 treatment) according to the manufacture instruction. * p<0.05, compared to control.

Measurement of Rap1 activity in porcine CASMCs (Fig 2A–2C) revealed that G-1(1 μM) and 007 treatments significantly increased Rap1 activity. In Fig 2A, the results showed that the stimulation of Rap1 activity by G-1 at 2.5 min and 5 min was similar to the stimulation effect of the selective Epac activator, 007 (50 μM) and the increased Rap1 activity induced by either G-1 or 007 were completely blocked by BFA (50 μM) (Fig 2A & 2B). In the presence of PGF2α, however, the activity of Rap1 did not change (Fig 2C). Together, these results suggest that as a downstream of Epac, Rap1 is involved in GPER-mediated coronary artery relaxation response.

**Fig 3:**
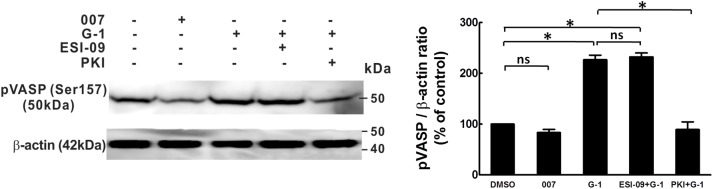
G-1 increases phosphorylation of VASP. Porcine coronary artery SMCs (passage 4 and 5) were serum deprived for 24 hours and then treated with: 0.01% DMSO as solvent control; 007 (50 μM); G-1 (1 μM); G-1(1 μM) + ESI-09 (10 μM) and PKI (5 μM). Left panel is a representative of Western blot for p-VASP and β–actin of three individual experiments. Right panel is a summary bar graph of the Western blot band densitometry analysis. Results are expressed as mean ± SE, * p<0.05, compared to the solvent control.

Vasodilator-stimulated phosphoprotein (VASP) can be phosphorylated by PKA at Ser 157, thus reflecting PKA activity [14]. G-1 (1 μM) increased p-VASP in porcine CASMCs, which was totally blocked by PKI but not by ESI-09, an Epac inhibitor [21] (Fig 3). Additionally, 007 had no significant effect on p-VASP compared with vehicle control treatment. Taken together, these data suggest that Epac and PKA are two discrete downstream targets of GPER and with only the PKA signaling pathways contributing to the phosphorylation of VASP.

### RhoA activity

**Fig 4:**
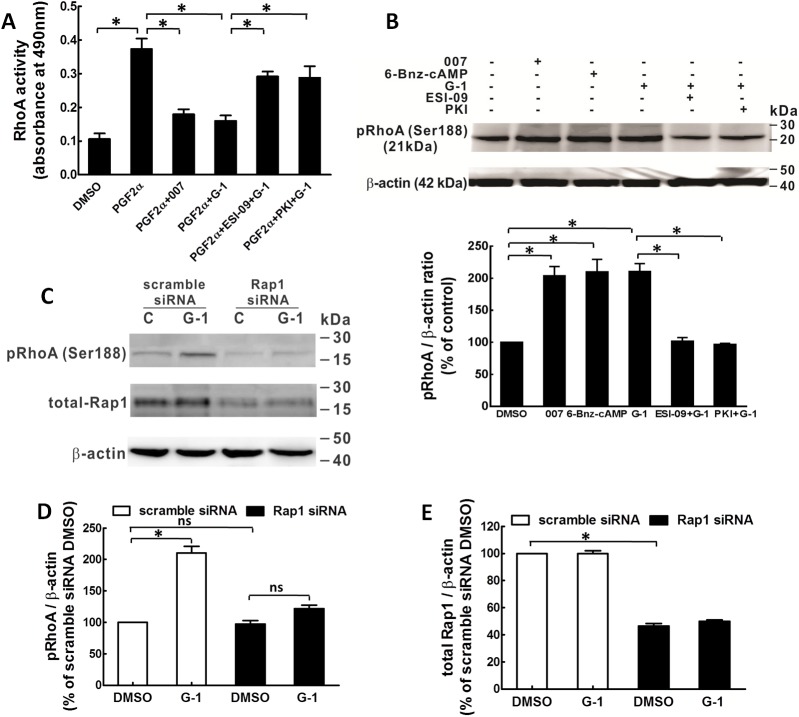
Epac and Rap1 are involved in the G-1-induced phosphorylation of RhoA and the inhibition of RhoA activity in porcine CASMCs. CASMCs were serum deprived for 18 hours before being treated with different drugs. A: RhoA activity in CASMCs was evaluated in cells treated for 2.5 min with 0.1% DMSO, serum, serum + 007 (100 μM), serum + G-1 (100 nM), 10% serum + ESI-09 (10 μM) + G-1 (100 nM) (n = 3), * p<0.05, compared to groups as indicated. B: Western blot detection of phosphorylation of RhoA at Ser188 in CASMCs. Cells were treated for 10 minutes with: 0.1% DMSO as solvent control; 007 (100 μM); 6-Bnz-cAMP (10 μM); G-1 (1 μM); ESI-09 (10 μM) + G-1 (1 μM) and PKI (5 μM) + G-1 (1 μM) (n = 3). Upper panel: a representative Western blot phosphor-Ser188 RhoA and β-actin of three experiments. Lower panel: Bar graph of the quantitative data of the p-RhoA bands evaluated by densitometry. Sample protein amounts were normalized to β-actin which was employed as a control for protein loading, * p<0.05, compared to the groups as indicated. C: A representative phosphorylation of RhoA and siRNA knock-down of Rap1 were detected by Western blots of three independent experiments. After transfected with either scramble siRNA or Rap1 siRNA for 48 hours, CASMCs were serum deprived for 24 hours and then treated in the presence or absence of G-1 (1 μM). β-actin was served as loading control. D&E: Bar graphs showing the summary data of p-RhoA and total Rap1 knock-down normalized to total β-actin respectively, the bands were evaluated by densitometry, * p<0.05, compared to the group as indicated in the graphs.

Previously we have shown that G-1 inhibits RhoA activity with a significant contribution of PKA to this inhibition [10]. In the current study, we measured RhoA activity and phosphorylation at Ser 188 (p-RhoA), which is inactivated state of RhoA [23], by treating porcine CASMCs with 007 and ESI-09 to test whether Epac also plays a role in GPER-mediated inhibition of RhoA activity. The results showed that 007 (50 μM) inhibited RhoA activity to a similar extent as G-1 (1 μM) and ESI-09 (10 μM) significantly inhibited the effect of G-1 (Fig 4A). The Epac effect on p-RhoA was examined by Western blot and the results showed that both 007 (50 μM) and the PKA agonist 6-Benz-cAMP (50 μM) increased p-RhoA similarly as that of G-1 (1 μM). The G-1 effect of increasing p-RhoA was significantly inhibited by either PKI or ESI-09 (Fig 4B). Furthermore, siRNA-mediated knock down of Rap1 significantly inhibited G-1-induced p-RhoA (Fig 4C & 4D). The efficiency of the Rap1 siRNA transfection was examined to be significant compared to the control group transfected with scramble siRNA (Fig 4E). Together, these results suggest that GPER activation inhibits RhoA activity through phosphorylation of RhoA via both Epac and PKA signaling.

### Phosphorylation of MLCP/MLC

**Fig 5:**
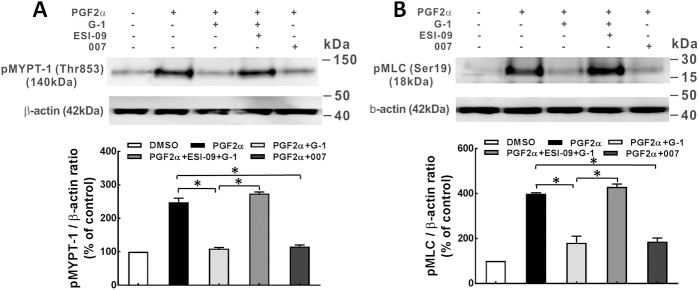
Epac is involved in the G-1 activation of MLCP in porcine coronary arteries. A: Western blot detection of phosphorylation of MLCP at the regulatory subunit, myosin-targeting subunit protein-1 (pMYPT1 at Thr853) in porcine coronary artery rings. Rings in isometric tension studies were incubated with DMSO (solvent control, 0.1%); PGF2α (1 μM); PGF2α (1 μM) + G-1 (1 μM); PGF2α (1 μM) + ESI-09 (10 μM) +G-1 (1 μM); and PGF2α (1 μM) + 007 (100 μM). Upper panel: A representative Western blot for p-MYPT1 from three individual experiments. Lower panel: Bar graph of the quantitative data of the Western blot bands evaluated by densitometry. Tissue sample protein amounts were normalized to β-actin which was employed as a control for protein loading, * p<0.05, compared between groups as indicated. B: Upper panel: the representative p-MLC detection by Western blot from three independent experiments. Porcine CASMCs were serum deprived for 24 hours, and then treated with drugs as in porcine coronaries, except that the concentration of ESI-09 was 10 μM. Lower panel: Bar graph showing the summary data of p-MLC which was normalized to total β-actin, the bands were evaluated by densitometry, * p<0.05, compared to the group as indicated.

Increased RhoA activity leads to phosphorylation of MYPT1 at Thr-853 via Rho kinase [24, 25], which inhibits the activity of MLCP and thus, results in increased MLC phosphorylation. Western blot analysis revealed that G-1 (1 μM) inhibited PGF2α (1 μM)-induced phosphorylation of MYPT1 at Thr-853 and subsequently, reduced phosphorylation of MLC, in both coronary artery rings and porcine CASMCs (Fig 5). Furthermore, the inhibitory effect of G-1 was partially reversed by each ESI-09 (10 μM). The Epac activator, 007 (50 μM), exerted a similar inhibitory effect on the phosphorylation of MYPT1 at Thr-853 and MLC as that of G-1 (1 μM), suggesting that Epac signaling is involved in the GPER-mediated inhibition of p-MYPT1 and p-MLC.

**Fig 6:**
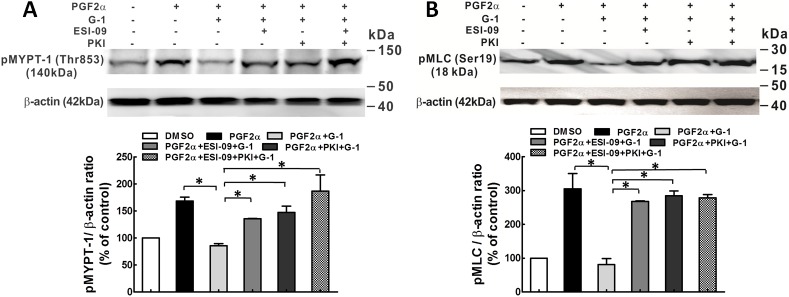
Epac and PKA exert additive effect on G-1-induced activation of MLCP in porcine coronary arteries. A: Western blot detection of pMYPT1 at Thr853 in artery rings. Artery rings were treated with: DMSO (solvent control, 0.1%); PGF2α (1 μM); PGF2α (1 μM) + G-1 (1 μM); PGF2α (1 μM) + ESI-09 (10 μM) +G-1 (1 μM); PGF2α (1 μM) + PKI (5 μM) + G-1 (1 μM); and PGF2α (1 μM) + ESI-09 (10 μM) + PKI (5 μM) + G-1 (1 μM). Upper panel: A representative Western blot for p-MYPT1 from three individual experiments. Lower panel: Bar graph summary of the quantitative data of the Western blot p-MYPT1 bands evaluated by densitometry. Tissue sample protein amounts were normalized to β-actin. B: Upper panel: A representative p-MLC detection by Western blots of three independent experiments. CASMCs were serum deprived for 24 hours, and then incubated with the drugs as used in arterial rings, except that 10 μM of ESI-09 was used rather than 25 μM. Lower panel: Bar graph showing the summary data of p-MLC normalized to total β-actin, the bands were evaluated by densitometry, * p<0.05, compared to the group as indicated in the graph.

In another set of experiments, both Epac and PKA antagonists were used in examining the role of Epac as well as PKA in the inhibitory effect of G-1 on the phosphorylation of MYPT1 and MLC. When added separately, ESI-09 (10 μM) as well as PKI (5 μM) partially reversed the inhibitory effect of G-1 on phosphorylation of MYPT1 and MLC; however, when both of ESI-09 (10 μM) and PKI (5 μM) were present, the G-1 -induced inhibition of the phosphorylation of MYPT1 and MLC was completely reversed (Fig 6). These results suggest that Epac/Rap1 signaling along with PKA is involved in the GPER-mediated inhibition of the phosphorylation of MLCP/MLC.

## Discussion

There is a growing interest in the role of GPER in cardiovascular regulation. The current work is the first to define the cAMP/ Epac signaling pathway in GPER-mediated coronary artery relaxation, which is mediated through attenuation of phosphorylation of MLCP and MLC via Rap1 inhibition of RhoA activity in parallel with PKA.

GPER activation induces relaxation in a variety of vascular beds [4, 5, 26, 27] and reduces tissue damage after cardiac ischemia-reperfusion or stroke [2, 3]. However, our understanding of the mechanisms that underlie GPER-mediated vasorelaxation has just begun to increase. Our recent work has shown that: 1) G-1 treatment of porcine CASMCs activates adenylyl cyclase (AC) and increases cAMP production in a concentration-dependent manner; 2) blocking of AC inhibits G-1 -induced porcine coronary artery relaxation in isometric tension studies, suggesting cAMP was involved in the GPER-mediated coronary artery relaxation; 3) downstream PKA signaling plays a role in GPER-mediated coronary relaxation by activating MLCP via inhibition of RhoA pathway [10]. In the current studies, we have found that, along with PKA, Epac/Rap1 signaling is also involved in the GPER-mediated vasorelaxation via inhibition of RhoA pathway.

The elevation in intracellular concentration of cAMP following activation of Gs-protein-coupled receptors is sufficient to activate Epac [12], a novel mechanism in the regulation of cardiovascular function [11]. Two isoforms, Epac1 and Epac2, have been identified [11]. Epac1 is the predominant isoform found in vascular smooth muscle of rat aorta and mesenteric artery [28]. Activation of Epac1 induces relaxation of adrenaline-contracted rat aortae [29] and phenylephrine-contracted rat mesenteric arteries [14]. In the present study, G-1 induced porcine coronary artery relaxation was significantly attenuated by the ARF-GEF inhibitor, brefeldin A (50 μM), which has been shown to be an Epac inhibitor [14]; furthermore, the Epac specific agonist, 8CPT-2Me-cAMP (007), relaxed arteries similar to that observed with G-1. Together these findings suggest that Epac, a downstream effector of cAMP, is also involved in the GPER-mediated relaxation effect; and functions similarly to PKA in mediating GPER vasorelaxation as our previous study reported [10].

Epac is the best described guanine nucleotide exchange factor (GEF) in the cardiovascular system and facilitates Rap1 cycling from the (GDP-bound) inactive state to the (GTP- bound) active state [30]. Rap1 isoforms, as members of the Ras family, are involved in the network of multiple proteins to regulate vascular endothelial cell proliferation, migration and endothelial permeability as well as vascular tone [31]. Rap1b in both smooth muscle and endothelium plays a key role in maintaining blood pressure [32, 33]. Rap1b−/− mice developed increased basal tone and hypertension that was potentially caused by: 1) increased contractility of smooth muscle to contractile agents, such as thromboxane, angiotensin II or phenylephrine; 2) defective endothelial release of dilatory nitric oxide in response to elevated blood flow [32, 33]. We tested the effect of GPER activation on Rap1 activity in porcine CASMC and observed that G-1 (1 μM) markedly increased Rap1 activity between 2 and 5 min, to a similar extent as 007; an increase that was blocked by BFA (50 μM), suggesting that GPER activation stimulates Rap1 activity via Epac in CASMC. Furthermore, our results showed that Rap1 siRNA transfection significantly inhibited G-1-induced phosphorylation of RhoA at serine 188, further validating the role of Rap1 in GPER-mediated signaling. Other in vitro studies have revealed several underlying mechanisms for Epac/Rap1 induced vascular relaxation, including attenuation of RhoA activity [14] and indirect modulation of K^+^ channel activity [20, 28].

Increase in RhoA activity stimulated by various vasoconstrictors leads to Rho kinase-mediated phosphorylation of MYPT1, at Thr-853, not at Thr-696 [24, 25]. The elevated phosphorylation of MYPT1 largely inhibits MLCP activity, therefore, maintaining MLC phosphorylation and thus vascular smooth muscle contraction. On the other hand, when MYPT1 is dephosphorylated, MLCP dephosphorylates MLC and induces relaxation [34]. Evidence from the work of other investigators, as well as our data suggest that cAMP/PKA signaling may inhibit RhoA activity by phosphorylating RhoA at Ser188, RhoA phosphorylation reduces the inhibitory effect of RhoA/Rho kinase on MLCP, thus allows MLCP to dephosphorylate MLC and cause relaxation of vascular smooth muscle independent of intracellular Ca^2+^ level [10, 23, 35]. Since the phosphorylation of MYPT1 at Thr-696 site is often spontaneously phosphorylated under resting conditions and is insensitive to stimuli by most agonists [25, 34], we studied only the phosphorylation of Thr-853. Our results showed that: 1) Epac antagonist, ESI-09, significantly inhibited G-1-induced phosphorylation of RhoA at Ser-188 and restored G-1-reduced RhoA activity; 2) Epac agonist, 007, on the other hand, increased phosphorylation of RhoA and decreased RhoA activity, similar to that of G-1 and PKA agonist, 6-Benz-cAMP; 3) G-1-induced reduction in phosphorylation of Thr-853 of MYPT1, was partially reversed when either PKA or Epac antagonists were present, with an additive effect when both antagonists were present, completely restoring p-MYPT1 levels. Together these findings demonstrate that Epac plays a similar role as PKA in GPER-mediated coronary relaxation via inhibition of RhoA and p-MYPT1. Consistent with our data are findings in smooth muscle of rat aorta, gut, and airway, in which Epac agonist, 007, as well as the vasodilator PGI_2_ analog, cicaprost, increased Rap1 activity and decreased RhoA activity [14]. Activation of a plasma membrane-bound G protein-coupled receptor, TGR5, known as GPBAR1 (G protein-coupled bile acid receptor1), has also been reported to cause relaxation of gastric smooth muscle, which is mediated through inhibition of RhoA/Rho kinase pathway via cAMP/Epac-dependent stimulation of Rap1 [36].

In vascular smooth muscle cells, both PKA and Epac pathways are necessary for cell growth inhibition. Epac synergizes with PKA to mediate cAMP-induced cell growth arrest [37]. In cAMP-mediated vascular, gut and airway smooth muscle relaxation, Epac and PKA are involved independently in the downstream signaling [14, 36]. In this study, we showed that Epac and PKA exert additive effect in mediating GPER downstream signaling at the phosphorylation of MYPT1, although they signal in separate pathways as confirmed by the result that neither Epac agonist nor antagonist had any effect on the phosphorylation of VASP at Ser-157, a marker for monitoring PKA activity [14].

**Fig 7:**
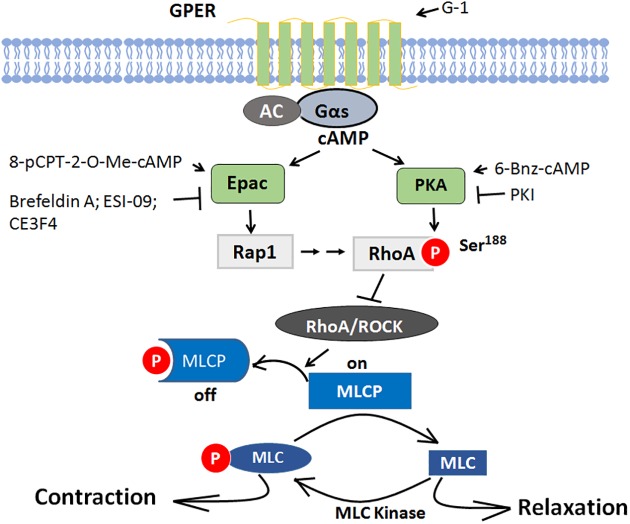
Proposed mechanism of Epac and PKA pathways in GPER-mediated porcine coronary artery relaxation signaling. When GPER is activated by G-1, it activates Gs protein. Gs stimulates adenlylyl cyclase (AC) and increases cAMP generation. Cyclic AMP activates both Epac and PKA, the downstream targets. Epac activates Rap1, then RhoA is phosphorylated at serine 188 by both Rap1 signaling and PKA, thereby the activity RhoA and its effector Rho Kinase (ROCK) is inhibited, Reduced Rho kinase activity removes its inhibitory effects on MLCP by decreasing the phosphorylation of MYPT1 (pThr853), the myosin phosphatase target subunit 1 of MLCP, leading to decreased phosphorylation of MLC20 and coronary artery relaxation.

In conclusion, the results of this study demonstrate that GPER-mediated porcine coronary relaxation involves both Epac and PKA signaling to inhibit the RhoA/ROCK effect on MLCP, and thereby, decrease phosphorylation of MLC and relaxation of coronary artery (Fig 7). These findings offer clearer understanding of the role of GPER in vascular tone regulation and provide a molecular basis for GPER as a potential drug target in preventing and treating cardiovascular disease both in women.

## References

[1] Han G, Li F, Yu X, White RE. GPER: a novel target for non-genomic estrogen action in the cardiovascular system. Pharmacol Res. 2013;71:53–60. 10.1016/j.phrs.2013.02.008 23466742

[2] Deschamps AM, Murphy E. Activation of a novel estrogen receptor, GPER, is cardioprotective in male and female rats. Am J Physiol Heart Circ Physiol. 2009;297(5):H1806–13. Epub 2009/09/01. PubMed Central PMCID: PMC2781389. 10.1152/ajpheart.00283.2009 19717735 PMC2781389

[3] Zhang B, Subramanian S, Dziennis S, Jia J, Uchida M, Akiyoshi K, et al Estradiol and G1 reduce infarct size and improve immunosuppression after experimental stroke. J Immunol. 2010;184(8):4087–94. Epub 2010/03/23. PubMed Central PMCID: PMC3142781. 10.4049/jimmunol.0902339 20304826 PMC3142781

[4] Haas E, Bhattacharya I, Brailoiu E, Damjanovic M, Brailoiu GC, Gao X, et al Regulatory role of G protein-coupled estrogen receptor for vascular function and obesity. Circulation research. 2009;104(3):288–91. Epub 2009/01/31. PubMed Central PMCID: PMC2782532. 10.1161/CIRCRESAHA.108.190892 19179659 PMC2782532

[5] Lindsey SH, Cohen JA, Brosnihan KB, Gallagher PE, Chappell MC. Chronic treatment with the G protein-coupled receptor 30 agonist G-1 decreases blood pressure in ovariectomized mRen2.Lewis rats. Endocrinology. 2009;150(8):3753–8. Epub 2009/04/18. PubMed Central PMCID: PMC2717873. 10.1210/en.2008-1664 19372194 PMC2717873

[6] Yu X, Ma H, Barman SA, Liu AT, Sellers M, Stallone JN, et al Activation of G protein-coupled estrogen receptor induces endothelium-independent relaxation of coronary artery smooth muscle. American journal of physiology Endocrinology and metabolism. 301(5):E882–8. Epub 2011/07/28. 10.1152/ajpendo.00037.2011 21791623 PMC3213995

[7] Meyer MR, Baretella O, Prossnitz ER, Barton M. Dilation of epicardial coronary arteries by the G protein-coupled estrogen receptor agonists G-1 and ICI 182,780. Pharmacology. 86(1):58–64. Epub 2010/07/20. 10.1159/000315497 20639684 PMC3201835

[8] Han G, Ma H, Chintala R, Fulton DJ, Barman SA, White RE. Essential role of the 90-kilodalton heat shock protein in mediating nongenomic estrogen signaling in coronary artery smooth muscle. The Journal of pharmacology and experimental therapeutics. 2009;329(3):850–5. Epub 2009/03/19. PubMed Central PMCID: PMC2683768. 10.1124/jpet.108.149112 19293389 PMC2683768

[9] Filardo E, Quinn J, Pang Y, Graeber C, Shaw S, Dong J, et al Activation of the novel estrogen receptor G protein-coupled receptor 30 (GPR30) at the plasma membrane. Endocrinology. 2007;148(7):3236–45. Epub 2007/03/24. 10.1210/en.2006-1605 17379646

[10] Yu X, Li F, Klussmann E, Stallone JN, Han G. G protein-coupled estrogen receptor 1 mediates relaxation of coronary arteries via cAMP/PKA-dependent activation of MLCP. American journal of physiology Endocrinology and metabolism. 2014;307(4):E398–407. 10.1152/ajpendo.00534.2013 25005496

[11] Metrich M, Berthouze M, Morel E, Crozatier B, Gomez AM, Lezoualc'h F. Role of the cAMP-binding protein Epac in cardiovascular physiology and pathophysiology. Pflugers Arch. 459(4):535–46. Epub 2009/10/27. 10.1007/s00424-009-0747-y 19855995

[12] Roberts OL, Dart C. cAMP signalling in the vasculature: the role of Epac (exchange protein directly activated by cAMP). Biochemical Society transactions. 2014;42(1):89–97. 10.1042/BST20130253 24450633

[13] Dao KK, Teigen K, Kopperud R, Hodneland E, Schwede F, Christensen AE, et al Epac1 and cAMP-dependent protein kinase holoenzyme have similar cAMP affinity, but their cAMP domains have distinct structural features and cyclic nucleotide recognition. The Journal of biological chemistry. 2006;281(30):21500–11. Epub 2006/05/27. 10.1074/jbc.M603116200 16728394

[14] Zieba BJ, Artamonov MV, Jin L, Momotani K, Ho R, Franke AS, et al The cAMP-responsive Rap1 guanine nucleotide exchange factor, Epac, induces smooth muscle relaxation by down-regulation of RhoA activity. The Journal of biological chemistry. 286(19):16681–92. Epub 2011/04/02. PubMed Central PMCID: PMC3089510. 10.1074/jbc.M110.205062 21454546 PMC3089510

[15] White RE, Han G, Maunz M, Dimitropoulou C, El-Mowafy AM, Barlow RS, et al Endothelium-independent effect of estrogen on Ca(2+)-activated K(+) channels in human coronary artery smooth muscle cells. Cardiovascular research. 2002;53(3):650–61. 11861036 10.1016/s0008-6363(01)00428-x

[16] White RE, Han G, Dimitropoulou C, Zhu S, Miyake K, Fulton D, et al Estrogen-induced contraction of coronary arteries is mediated by superoxide generated in vascular smooth muscle. American journal of physiology Heart and circulatory physiology. 2005;289(4):H1468–75. PubMed Central PMCID: PMC1380187. 10.1152/ajpheart.01173.2004 16162867 PMC1380187

[17] Li F, Yu X, Szynkarski CK, Meng C, Zhou B, Barhoumi R, et al Activation of GPER Induces Differentiation and Inhibition of Coronary Artery Smooth Muscle Cell Proliferation. PLoS One. 2013;8(6):e64771 PubMed Central PMCID: PMC3686788. 10.1371/journal.pone.0064771 23840305 PMC3686788

[18] Calderon-Sanchez E, Delgado C, Ruiz-Hurtado G, Dominguez-Rodriguez A, Cachofeiro V, Rodriguez-Moyano M, et al Urocortin induces positive inotropic effect in rat heart. Cardiovasc Res. 2009;83(4):717–25. 10.1093/cvr/cvp161 19460778

[19] Nini L, Dagnino L. Accurate and reproducible measurements of RhoA activation in small samples of primary cells. Analytical biochemistry. 2010;398(1):135–7. 10.1016/j.ab.2009.11.011 19917260

[20] Roberts OL, Kamishima T, Barrett-Jolley R, Quayle JM, Dart C. Exchange protein activated by cAMP (Epac) induces vascular relaxation by activating Ca2+-sensitive K+ channels in rat mesenteric artery. The Journal of physiology. 2013;591(Pt 20):5107–23. PubMed Central PMCID: PMC3810813. 23959673 10.1113/jphysiol.2013.262006 PMC3810813

[21] Zhu Y, Chen H, Boulton S, Mei F, Ye N, Melacini G, et al Biochemical and pharmacological characterizations of ESI-09 based EPAC inhibitors: defining the ESI-09 "therapeutic window". Scientific reports. 2015;5:9344 PubMed Central PMCID: PMCPMC4366844. 10.1038/srep09344 25791905 PMC4366844

[22] Courilleau D, Bisserier M, Jullian JC, Lucas A, Bouyssou P, Fischmeister R, et al Identification of a tetrahydroquinoline analog as a pharmacological inhibitor of the cAMP-binding protein Epac. The Journal of biological chemistry. 2012;287(53):44192–202. PubMed Central PMCID: PMCPMC3531735. 10.1074/jbc.M112.422956 23139415 PMC3531735

[23] Guilluy C, Rolli-Derkinderen M, Loufrani L, Bourge A, Henrion D, Sabourin L, et al Ste20-related kinase SLK phosphorylates Ser188 of RhoA to induce vasodilation in response to angiotensin II Type 2 receptor activation. Circulation research. 2008;102(10):1265–74. 10.1161/CIRCRESAHA.107.164764 18420945

[24] Dimopoulos GJ, Semba S, Kitazawa K, Eto M, Kitazawa T. Ca2+-dependent rapid Ca2+ sensitization of contraction in arterial smooth muscle. Circulation research. 2007;100(1):121–9. PubMed Central PMCID: PMC2212616. 10.1161/01.RES.0000253902.90489.df 17158339 PMC2212616

[25] Kitazawa T, Eto M, Woodsome TP, Khalequzzaman M. Phosphorylation of the myosin phosphatase targeting subunit and CPI-17 during Ca2+ sensitization in rabbit smooth muscle. The Journal of physiology. 2003;546(Pt 3):879–89. PubMed Central PMCID: PMC2342583. 10.1113/jphysiol.2002.029306 12563012 PMC2342583

[26] Yu X, Ma H, Barman SA, Liu AT, Sellers M, Stallone JN, et al Activation of G protein-coupled estrogen receptor induces endothelium-independent relaxation of coronary artery smooth muscle. American journal of physiology Endocrinology and metabolism. 2011;301(5):E882–8. Epub 2011/07/28. PubMed Central PMCID: PMC3213995. 10.1152/ajpendo.00037.2011 21791623 PMC3213995

[27] Meyer MR, Haas E, Prossnitz ER, Barton M. Non-genomic regulation of vascular cell function and growth by estrogen. Mol Cell Endocrinol. 2009;308(1–2):9–16. Epub 2009/06/25. PubMed Central PMCID: PMC2780565. 10.1016/j.mce.2009.03.009 19549587 PMC2780565

[28] Purves GI, Kamishima T, Davies LM, Quayle JM, Dart C. Exchange protein activated by cAMP (Epac) mediates cAMP-dependent but protein kinase A-insensitive modulation of vascular ATP-sensitive potassium channels. The Journal of physiology. 2009;587(Pt 14):3639–50. Epub 2009/06/06. PubMed Central PMCID: PMC2742287. 10.1113/jphysiol.2009.173534 19491242 PMC2742287

[29] Sukhanova IF, Kozhevnikova LM, Popov EG, Podmareva ON, Avdonin PV. Activators of Epac proteins induce relaxation of isolated rat aorta. Doklady biological sciences: proceedings of the Academy of Sciences of the USSR, Biological sciences sections / translated from Russian. 2006;411:441–4. 10.1134/s0012496606060044 17425034

[30] Jeyaraj SC, Unger NT, Chotani MA. Rap1 GTPases: an emerging role in the cardiovasculature. Life Sci. 88(15–16):645–52. Epub 2011/02/08. PubMed Central PMCID: PMC3090149. 10.1016/j.lfs.2011.01.023 21295042 PMC3090149

[31] Chrzanowska-Wodnicka M. Distinct functions for Rap1 signaling in vascular morphogenesis and dysfunction. Experimental cell research. 2013;319(15):2350–9. PubMed Central PMCID: PMC3913003. 10.1016/j.yexcr.2013.07.022 23911990 PMC3913003

[32] Lakshmikanthan S, Zieba BJ, Ge ZD, Momotani K, Zheng X, Lund H, et al Rap1b in smooth muscle and endothelium is required for maintenance of vascular tone and normal blood pressure. Arteriosclerosis, thrombosis, and vascular biology. 2014;34(7):1486–94. 10.1161/ATVBAHA.114.303678 24790136 PMC4224284

[33] Lakshmikanthan S, Zheng X, Nishijima Y, Sobczak M, Szabo A, Vasquez-Vivar J, et al Rap1 promotes endothelial mechanosensing complex formation, NO release and normal endothelial function. EMBO Rep. 2015;16(5):628–37. PubMed Central PMCID: PMCPMC4428051. 10.15252/embr.201439846 25807985 PMC4428051

[34] Khromov A, Choudhury N, Stevenson AS, Somlyo AV, Eto M. Phosphorylation-dependent autoinhibition of myosin light chain phosphatase accounts for Ca2+ sensitization force of smooth muscle contraction. The Journal of biological chemistry. 2009;284(32):21569–79. Epub 2009/06/18. PubMed Central PMCID: PMC2755881. 10.1074/jbc.M109.019729 19531490 PMC2755881

[35] Puetz S, Lubomirov LT, Pfitzer G. Regulation of smooth muscle contraction by small GTPases. Physiology. 2009;24:342–56. 10.1152/physiol.00023.2009 19996365

[36] Rajagopal S, Kumar DP, Mahavadi S, Bhattacharya S, Zhou R, Corvera CU, et al Activation of G protein-coupled bile acid receptor, TGR5, induces smooth muscle relaxation via both Epac- and PKA-mediated inhibition of RhoA/Rho kinase pathway. American journal of physiology Gastrointestinal and liver physiology. 2013;304(5):G527–35. PubMed Central PMCID: PMC3602680. 10.1152/ajpgi.00388.2012 23275618 PMC3602680

[37] Hewer RC, Sala-Newby GB, Wu YJ, Newby AC, Bond M. PKA and Epac synergistically inhibit smooth muscle cell proliferation. Journal of molecular and cellular cardiology. 2011;50(1):87–98. PubMed Central PMCID: PMC3093616. 10.1016/j.yjmcc.2010.10.010 20971121 PMC3093616

